# Editorial: Weighing the impact of being overweight on female reproductive function and fertility

**DOI:** 10.3389/frph.2025.1554284

**Published:** 2025-02-03

**Authors:** Theocharis Koufakis, Dimitrios Patoulias, Kulvinder Kochar Kaur, Djordje S. Popovic

**Affiliations:** ^1^ Second Propedeutic Department of Internal Medicine, Hippokration General Hospital, Aristotle University of Thessaloniki, Thessaloniki, Greece; ^2^ Kulvinder Kaur Centre for Human Reproduction, Jalandhar, India; ^3^ Clinic for Endocrinology, Diabetes and Metabolic Disorders, Clinical Centre of Vojvodina, Medical Faculty, University of Novi Sad, Novi Sad, Serbia

**Keywords:** obesity, overweight, infertility, polycystic ovarian syndrome, weight loss

**Editorial on the Research Topic**
Weighing the impact of being overweight on female reproductive function and fertility

The prevalence of overweight and obesity has followed a growing trend in recent decades worldwide (1). For many years, obesity has been considered primarily an aesthetic problem, rather than a chronic, serious, and relapsing disease. Fortunately, the medical community is increasingly acknowledging that overweight and obesity are associated with severe complications, including cardiometabolic disorders, chronic kidney disease, cognitive decline, malignancies, obstructive sleep apnea, urinary incontinence, osteoarthritis, and depression, among others (2, 3).

However, the link between obesity and female infertility is probably one of the most underestimated aspects of increased adiposity. Epidemiological data suggest a three-fold higher risk of infertility in women living with obesity compared to women with normal body weight (4). Although both overweight and obesity have been associated with a lower probability of conception, the risk becomes significantly greater as the body mass index (BMI) increases (5). Available studies with long follow-up suggest that childhood obesity contributes to an increased risk of infertility later in life (6). Given the high prevalence of overweight/obesity in developing parts of the world, the problem affects women in both high- and low-income countries (7); however, limited resources in the latter make the challenge even greater.

Insulin resistance, inflammation, coagulation abnormalities, polycystic ovary syndrome (PCOS), and disturbances in oocyte differentiation and maturation have been proposed as potential links between the two disorders (8). Women with obesity may have elevated androgen levels even in the absence of PCOS, resulting in reduced concentrations of luteinizing hormone (9). In some cases, obesity itself is the main source of causal mechanisms, while in others, it simply represents a mediator between infertility and a cascade of other disorders that negatively affect women's health. However, several pathophysiological aspects of this bidirectional relationship remain poorly understood. Interestingly, weight reduction has been shown to improve the probability of conception (10), underlining the need to effectively implement weight loss interventions to improve relevant outcomes.

The present research topic includes cutting-edge research that aims to elucidate the association between female reproductive disorders and obesity. Sun et al. provided evidence that depression mediates the relationship between weight-adjusted waist index and secondary infertility, highlighting the complex interactions between several factors that contribute to impaired fertility in women with overweight and obesity. Vedelek et al. showed that in women receiving *in vitro* fertilization treatment, reproduction hormone levels exhibit a decreasing trend with increasing BMI, supporting an evolutionary selective role of nutritional status. Zhang et al. identified an altered lipid profile in the follicular fluid of women with PCOS and insulin resistance compared to controls. These data could prove useful for the future use of plasmalogens as biomarkers of infertility in PCOS. Gitsi et al. reviewed current evidence on the role of nutrition and exercise in improving conception rates in women with obesity, underlining the need for a broad implementation of lifestyle interventions in the treatment of reproductive disorders. In a Mendelian randomization study, Xu et al. use data from the NHANES cohort and suggest an association between depression and infertility in women with obesity, underscoring the importance of incorporating mental health and weight management strategies into infertility treatment protocols. In a similar study design, Zhang et al. identified three protein-coding genes (*CDCP1, GLRX2*, and *KIRREL2*) as genetic risk factors for PCOS, shedding light on the pathophysiological abnormalities of the syndrome. A systematic review and meta-analysis by Ren et al. that included 25 randomized controlled trials and data from 1,636 women with PCOS and overweight/obesity showed that various nutritional supplements could exert beneficial effects on markers of oxidative stress, insulin resistance, and hormonal profile.

The future comes with important challenges in the field of the association between female reproductive health and obesity. These include better understanding of the underlying pathogenetic mechanisms, clearer characterization of relevant risk factors, identification of prognostic biomarkers, and evaluation of the efficacy and safety of new anti-obesity drugs, as well as metabolic surgery, in fertility outcomes through well-designed research. Until all these data become available, increased vigilance from health professionals, but also policy makers and societies, is required to effectively recognize overweight and obesity as important predisposing factors to female infertility and provide women affected with evidence-based care and support.
